# *FAM3D* as a Prognostic Indicator of Head and Neck Squamous Cell Carcinoma Is Associated with Immune Infiltration

**DOI:** 10.1155/2022/5851755

**Published:** 2022-12-03

**Authors:** Lizhu Chen, Jie Lin, Bin Lan, Jiani Xiong, Yaoming Wen, Yu Chen, Chuan-ben Chen

**Affiliations:** ^1^Department of Medical Oncology, Clinical Oncology School of Fujian Medical University, Fujian Cancer Hospital, Fuzhou, Fujian Province, China; ^2^Cancer Bio-immunotherapy Center, Clinical Oncology School of Fujian Medical University and Fujian Cancer Hospital, Fuzhou, Fujian Province, China; ^3^Fujian Provincial Key Laboratory of Translational Cancer Medicine, Fuzhou, Fujian Province, China; ^4^Departments of Gynecology, Clinical Oncology School of Fujian Medical University, Fujian Cancer Hospital, Fuzhou, Fujian Province, China; ^5^Fujian Institute of Microbiology, Fuzhou, Fujian Province, China; ^6^Department of Radiation Oncology, Clinical Oncology School of Fujian Medical University, Fujian Cancer Hospital, Fuzhou, Fujian Province, China

## Abstract

**Background:**

Globally, head and neck squamous cell carcinoma (HNSCC) is a common malignant tumor with high morbidity and mortality. Hence, it is important to find effective biomarkers for the diagnosis and prediction of the prognosis of patients with HNSCC. *FAM3D* had been proven to be vital in other cancers. However, its predictive and therapeutic value in HNSCC is unclear. Therefore, it is valuable to explore the association between the expression level of *FAM3D* and its impacts on the prognosis and tumor microenvironment in HNSCC.

**Methods:**

The Cancer Genome Atlas (TCGA) dataset, Genotype-Tissue Expression (GTEx) dataset, the Clinical Proteomic Tumor Analysis Consortium (CPTAC) dataset, and The Human Protein Atlas (THPA) website were used to assess HNSCC expressions in tumor and nontumor tissues. Then, we further conducted immunohistochemistry experiment as internal cohort to validate the same results. The Cox regression analysis, Kaplan–Meier analysis, and nomograms were performed to find the predictive prognostic value of *FAM3D* in HNSCC patients and its relationship with the clinicopathological features in HNSCC. The Gene Expression Omnibus (GEO) dataset was utilized to externally verify the prognosis value of *FAM3D* in HNSCC. Gene Set Enrichment Analysis (GESA) was applied to search the molecular and biological functions of *FAM3D*. The association between *FAM3D* and immune cell infiltration was investigated with the Tumor Immune Estimating Resource, version 2 (TIMER2). The relationships between *FAM3D* expression and tumor microenvironment (TME) scores, immune checkpoints, and antitumor compound half-maximal inhibitory concentration predictions were also explored.

**Results:**

In different datasets, *FAM3D* mRNA and protein levels were all significantly lower in HNSCC tissues than in normal tissues, and they were strongly inversely associated with tumor grade, stage, lymph node metastasis, and T stage. Patients with high-*FAM3D*-expression displayed better prognosis than those with low-*FAM3D*-expression. *FAM3D* was also determined to be a suitable biomarker for predicting the prognosis of patients with HNSCC. This was externally validated in the GEO dataset. As for gene and protein level, the functional and pathway research results of FAM3D indicated that it was enriched in alteration of immune-related pathways in HNSCC. The low-expression group had higher stromal and ESTIMATE scores by convention than the high-expression group. *FAM3D* expression were found to be positively correlated with immune infiltrating cells, such as cancer-associated fibroblasts, myeloid-derived suppressor cells, macrophage cells, T cell CD8+ cells, regulatory T cells, and T cell follicular helper cells. *FAM3D*'s relationships with immune checkpoints and sensitivity to antitumor drugs were also investigated.

**Conclusion:**

Our study explored the impact of *FAM3D* as a favorable prognostic marker for HNSCC on the tumor immune microenvironment from multiple perspectives. The results may provide new insights into HNSCC-targeted immunotherapy.

## 1. Introduction

HNSCC is a common malignant tumor worldwide which is originated in the mucosal epithelium of the oral cavity, pharynx, and larynx. Globally, in 2018, there were 830,000 new cases and 430,000 deaths related to head and neck cancer [1]. Of the total number of patients diagnosed with head and neck cancer, 70%–80% were diagnosed as locally advanced or advanced, and the 5-year survival rate of recurrent or metastatic HNSCC was only 3.6% [2]. Treatment is generally multimodal, consisting of surgery followed by chemoradiotherapy (CRT) for oral cavity cancers and primary CRT for pharynx and larynx cancers. Immunotherapy is also important in HNSCC treatment. Tumor cells evade immune surveillance by activating inhibitory checkpoint pathways and inhibiting antitumor T cell responses [3]. Immunotherapy has proven to be an effective strategy for the treatment of patients with HNSCC [4, 5]. The consensus guidelines of the United States Food and Drug Administration (FDA), the European Commission, and the Society for Immunotherapy of Cancer (SITC) serve as a foundation to guide clinicians' decisions of applying immunotherapies in recurrence of HNSCC [6]. Therefore, finding possible biomarkers for predicting the prognosis and immune statues of HNSCC are critical.

The proportion of regulatory T (Treg) cells in the peripheral blood of patients diagnosed with HNSCC was increased when compared with healthy people [7]. In turn, Treg cells express immune checkpoint receptors (e.g., CTLA-4 and PD-1) and immunosuppressive receptors (e.g., CD39 and transforming growth factor-1). This suggests that tumor cells begin to establish an immunosuppressive environment in patients with HNSCC. Currently, only a proportion of patients have achieved satisfactory results in immunotherapy. Thus, elucidating the altered tumor microenvironment under immunotherapy and finding effective biomarkers for targeted immunotherapy may provide new directions for the treatment of HNSCC.

Genetic instability and dysregulation of gene expression serve as the hallmarks of cancer are especially common in HNSCC. Tumor-associated antigens have been studied in these tumors accordingly [8, 9]. *FAM3D* is a cytokine-like family with a 4-helix bundle structure composed of *FAM3A*, *FAM3B*, *FAM3C*, and *FAM3D* 4 members. *FAM3D* is highly expressed in certain tissues (i.e., the placenta) and has an important role in cell proliferation [10]. *FAM3D* is extensively expressed in the gastrointestinal tract and has been linked to dietary control [11], lipid metabolism, and energy metabolism [12, 13]. *FAM3D* also acts as an agonist of the formyl peptide receptor [14], induces neutrophil recruitment through the G-coupled protein signaling pathway, and has proinflammatory properties [15]. Therefore, *FAM3D* is vital in gastrointestinal homeostasis, protection against inflammation-related cancers, and normal microbiota composition [16, 17].

However, there are few studies on *FAM3D* in HNSCC. This study explores the role of *FAM3D* in the diagnosis, prognosis, and tumor microenvironment (TME) scores in HNSCC via extensive bioinformatic data, which was crucial for the further development of immunotherapy for HNSCC.

## 2. Materials and Methods

### 2.1. Differential Gene and Protein Expression of *FAM3D* in HNSCC Tissues and Normal Tissues

For gene level, TIMER2 [18] web application (http://timer.cistrome.org/) was utilized to reveal the differential expressions of the *FAM3D* between tumor tissues and nearby normal tissues in all TCGA [19] tumors. Distributions of the gene expression levels were visualized via box plots. The number of stars indicated the statistical significance determined by the Wilcoxon test (^∗^: *p* < 0.05; ^∗∗^: *p* < 0.01; ^∗∗∗^: *p* < 0.001). The Gene Expression Profiling Interactive Analysis, version 2 (GEPIA2) web server (http://gepia2.cancer-pku.cn/#index) was used to generate box plots of the expression differences between HNSCC tissues and the matching normal tissues from TCGA and the Genotype-Tissue Expression (GTEx) databases. A *p* value threshold of 0.01, a log2 fold change (FC) cut-off of 1, and “Match TCGA normal and GTEx data” were established as the settings. The box plot was transformed using log2 (transcripts per million +1) expression data. Next, the RNA-Seq (STAR-Count) and clinical information data from the TCGA HNSCC dataset through the Genomic Data Commons (GDC) data portal were downloaded and preprocessed. The messenger RNA (mRNA) expression differences and pairing differences of *FAM3D* in HNSCC tissues and normal tissues were then calculated. The statistical analysis was performed using Rv.4.2.0 program. Statistical significance was determined at *p* < 0.05.

For protein level, the UALCAN [20] (University of Alabama at Birmingham Cancer) data analysis portal (http://ualcan.path.uab.edu/analysis-prot.html) was adopted for the protein expression analysis of CPTAC [21] dataset. The *Z* values represented standard deviations from the median across the samples for the given cancer type [21]. The log2 spectral count ratio values from the CPTAC were first normalized within each sample profile, then normalized across samples [21]. The data of protein expression with immunohistochemistry came from THPA (https://www.proteinatlas.org) [22].

In order to enhance the reliability of the results, immunohistochemistry experiment was then developed to identify the expression level of *FAM3D* in HNSCC and normal tissues. Tissue chips of HNSCC samples (HOraC080PG01) were acquired from Shanghai Outdo Biotech Company (Shanghai, China). The experimental procedures were performed as follows. First, formalin-fixed paraffin-embedded tissue chips were heated up by an oven. Paraffin sections were dewaxed by automatic dyeing machine (LEICAST5020, Leica). Second, antigen repair was then performed with an antigen repair apparatus (PT Link, Dako). Third, they were incubated overnight for 4°C after dripping with rabbit anti-*FAM3D* antibody (1 : 500, bs-14992R, Bioss). Forth, EnVision™ FLEX+ Mouse High pH (Link) (K8002, Dako) was utilized for the second antibody incubation. Fifth, slides were put into Dako automatic immunohistochemistry instrument. Last, they were stained with hematoxylin and sealed with neutral resin.

### 2.2. Survival Prognosis Analysis of *FAM3D* in HNSCC

For gene level, with the GEPIA2 application, this study obtained the overall survival (OS) plots of *FAM3D* for multiple tumors, including HNSCC. The hypothesis was tested using the log-rank test, and the survival map was created using GEPIA2's “survival analysis” module. The GDC and GEO databases (GSE65858) were used to extract *FAM3D* mRNA expression data and clinical information from HNSCC patients. The OS and progression-free survival (PFS) analyses were performed with the help of R packages (survival, survminer, and limma). For the segmentation of the high and low expression queues, high cut-off values (50%) and low cut-off values (50%) were employed as expression thresholds. For protein level, an association between *FAM3D* protein expression with the prognosis of HNSCC samples was obtained from the CPTAC dataset.

### 2.3. Correlation between *FAM3D* and Clinicopathological Features

In the TCGA cohort, the relevance between the clinicopathological variables and the *FAM3D* expression level was performed using logistic regression analysis. The predictive value of the expression level in the tumor tissues and normal tissues was tested by receiver operating characteristic (ROC) curves and the calculation of the area under the ROC curves (AUC). The R package “rms” was utilized to establish the nomograms and calibration plots. The above statistical analyses were all carried out by R v4.2.0; *p* < 0.05 was deemed statistically significant.

For gene level, in the GSE65858 cohort, the relevance between the clinicopathological variables and the *FAM3D* expression level was investigated using logistic regression analysis. The clinicopathological features of HNSCC and the impact of *FAM3D* expression on patient survival and prognosis were determined using univariate and multivariate Cox regression analyses. R v4.2.0 software was employed to conduct the statistical analysis. The connection between the OS and *FAM3D* expression was investigated using Kaplan–Meier and log-rank tests. Statistical significance was defined as a *p* < 0.05. For protein level, the relationship between *FAM3D* expression level and the clinical features in HNSCC patients was obtained from the CPTAC dataset.

### 2.4. Analysis of Coexpressed and Differentially Expressed Genes (DEGs)

This study evaluated the genes that were coexpressed with *FAM3D* and plotted the coexpression circle. According to the median expression level of *FAM3D*, the samples were listed as the high- and the low-expression group. To determine the coexpressed genes with *FAM3D*, the RNA sequencing data was performed using the limma and corrplot packages with the following parameters: corFilter = 0.6 and pFilter = 0.001 [23]. Then, an unpaired Student's *t*-test within the DESeq2 R program was carried out for further identification; ∣log 2FC | >1 and adjusted *p* < 0.05 were considered thresholds for the DEGs.

### 2.5. Functional and Pathway Enrichment Analysis

For gene level, in the TCGA cohort, clusterProfiler R software [24] was utilized to circulate the Kyoto Encyclopedia of Genes and Genomes (KEGG) pathway and the Gene Ontology (GO) analysis of *FAM3D* with the FDR (false discovery rate) cut-off of 0.05 [25]. These results were presented by the “ggplot2” package's bubble plots. For protein level, in the CPTAC cohort, the relationship between *FAM3D* protein expression and its pathways was obtained.

### 2.6. Tumor Microenvironment Analysis

The correlation between *FAM3D* expression and immune cell infiltration in HNSCC was investigated using the TIMER2 web application. The cancer-associated fibroblasts (CAFs), T cell CD8+ cells, Treg cells, Tfhs (T cell follicular helper cells), myeloid-derived suppressor cells (MDSCs), and macrophage cells were isolated. Estimates of CAFs and immune infiltration were made using the TIMER2, CIBERSORT, CIBERSORT-ABS, QUANTISEQ, XCELL, MCPCOUNTER, and EPIC algorithms. After adjusting for purity, the Spearman rank correlation test yielded the *p* and partial correlation values. A heat map and a scatter plot were used to visualize the data. Moreover, the link between the immune checkpoints and *FAM3D* was performed.

The estimation of stromal and immune cells in the malignant tumor tissues was analyzed in both the high- and low-*FAM3D*-expression groups using the expression data (ESTIMATE) scores, including the stromal and immune scores. Correlations between *FAM3D* expression and immune checkpoints were identified by Pearson's correlation coefficient. The data were visualized in a heat map through the “pheatmap” package.

### 2.7. Prediction of Treatment Response

The pRRophetic R package was performed to predict the drug response as indicated by the half-maximal inhibitory concentration (IC50) based on the Genomics of Drug Sensitivity in Cancer (GDSC) [26].

## 3. Results

### 3.1. *FAM3D* Demonstrated Downregulated Expression in HNSCC Tissues

**Figure 1:**
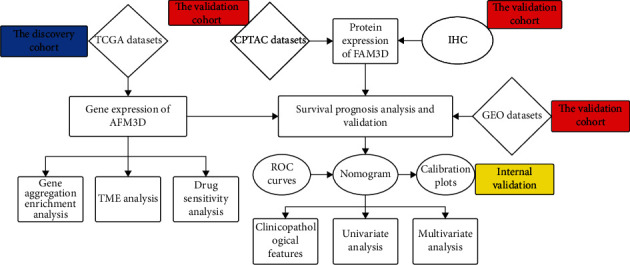
The study's flow chart.

**Figure 2:**
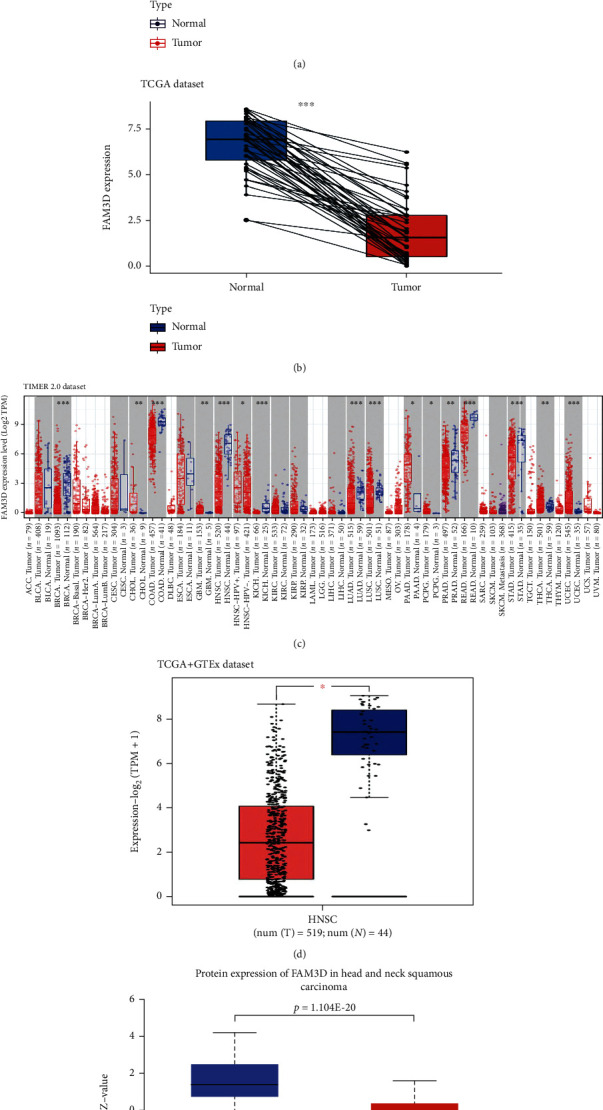
Expression level of *FAM3D* in HNSCC and normal tissues. (a) *FAM3D* expression in HNSCC and normal tissues from the TCGA dataset. (b) *FAM3D* expression in HNSCC and matched tumor-adjacent normal tissues from the TCGA dataset. (c) *FAM3D* expression in cancerous and normal tissues across different cancers based on the TIMER2 dataset; ^∗^*p* < 0.05; ^∗∗^*p* < 0.01; ^∗∗∗^*p* < 0.001. (d) *FAM3D* expression in cancerous and normal tissues from TCGA and GTE datasets. The box plot data were supplied; ^∗^*p* < 0.05. (e) Based on the CPTAC dataset, the *FAM3D* protein expression in HNSCC and normal tissues.

The flow of the study was exhibited in Figure 1. *FAM3D* mRNA expression was significantly lower in HNSCC tissues than in normal tissues in the TCGA dataset (Figure 2(a)). The paired difference analysis revealed the same result (Figure 2(b)). Additionally, data from the TIMER2 and GEPIA2 web applications both indicated that *FAM3D* expression levels were significantly lower in HNSCC tissues than in normal tissues (Figures 2(c) and 2(d)). To identify the above findings, the expression of the *FAM3D* protein in samples from the CPTAC database was evaluated. When compared with normal tissues, *FAM3D* protein expression was significantly lower in HNSCC tissues (Figure 2(e)).

**Figure 3:**
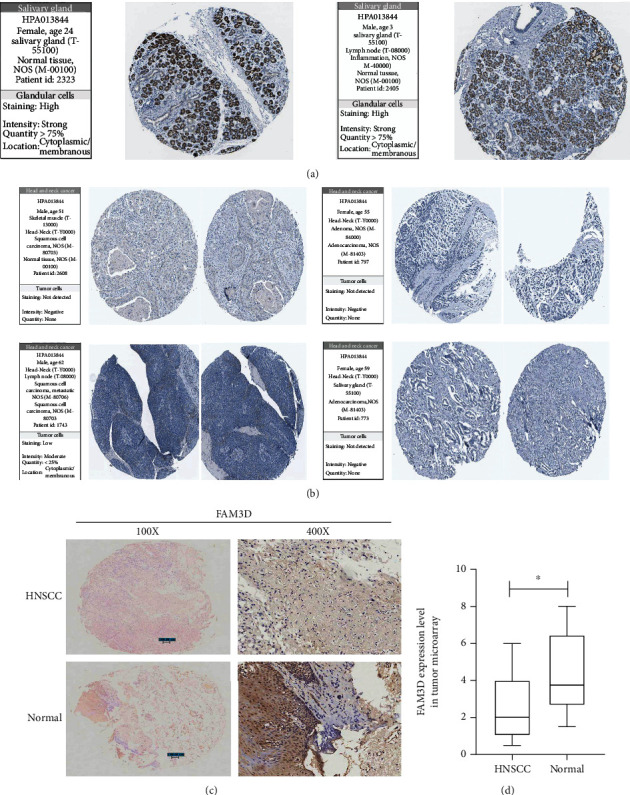
Protein expression level of *FAM3D* in HNSCC and normal tissues by immunohistochemical analysis. (a) *FAM3D* protein was highly expressed in the glandular cells of the salivary gland. (b) *FAM3D* protein was expressed in low amounts or not expressed in the tumor cells of HNSCC. (c) IHC staining of *FAM3D* expression from a tissue microarray of HNSCC patients (100×: scale bar, 100 *μ*m; 400×: scale bar, 50 *μ*m). (d). Mistoscores of *FAM3D* expression from the tissue microarray. HNSCC patients (num = 47) and normal tissues (num = 6).

Furthermore, the immunohistochemical results of *FAM3D* in HNSCC tumor tissues and normal tissues were examined from THPA. Protein intensity of *FAM3D* in normal tissues was strong (Figure 3(a)), while that in tumor tissues was negative or moderate (Figure 3(b)). The results of immunohistochemical experiment we conducted confirmed our previous conjecture (Figures 3(c) and 3(d)).

### 3.2. Survival Analysis of *FAM3D* in HNSCC

**Figure 4:**
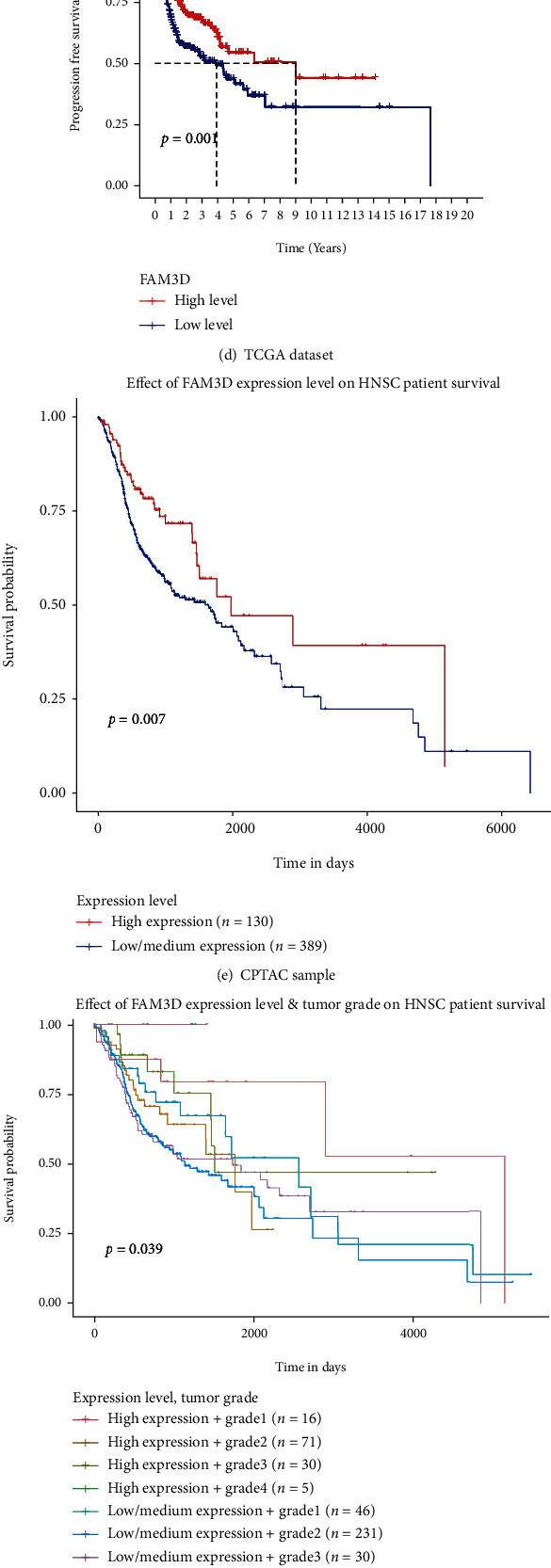
Correlation between *FAM3D* gene expression and survival prognosis of HNSCC. (a, b) The OS in different tumors in TCGA and GTEx datasets by *FAM3D* gene expression. (c, d) Kaplan–Meier curves of the OS and PFS of HNSCC patients in the TCGA database by *FAM3D* gene expression. (e, f) Kaplan–Meier curves of the OS of HNSCC patients by *FAM3D* protein expression in the CPTAC database. (g) Kaplan–Meier curves of the OS of HNSCC patients in the GEO database by *FAM3D* gene expression.

By data mining in TCGA cohort, GEPIA2 datasets and GEO cohort, we all found that HNSCC patients with higher *FAM3D* mRNA expressions displayed a better OS (Figures 4(a)–4(d) and 4(g)). And HNSCC patients with higher *FAM3D* protein expressions seem to have a better OS and PFS by CPTAC dataset (Figures 4(e) and 4(f)).

### 3.3. Association of *FAM3D* with Clinical Factors and Prognosis in TCGA Cohort

**Figure 5:**
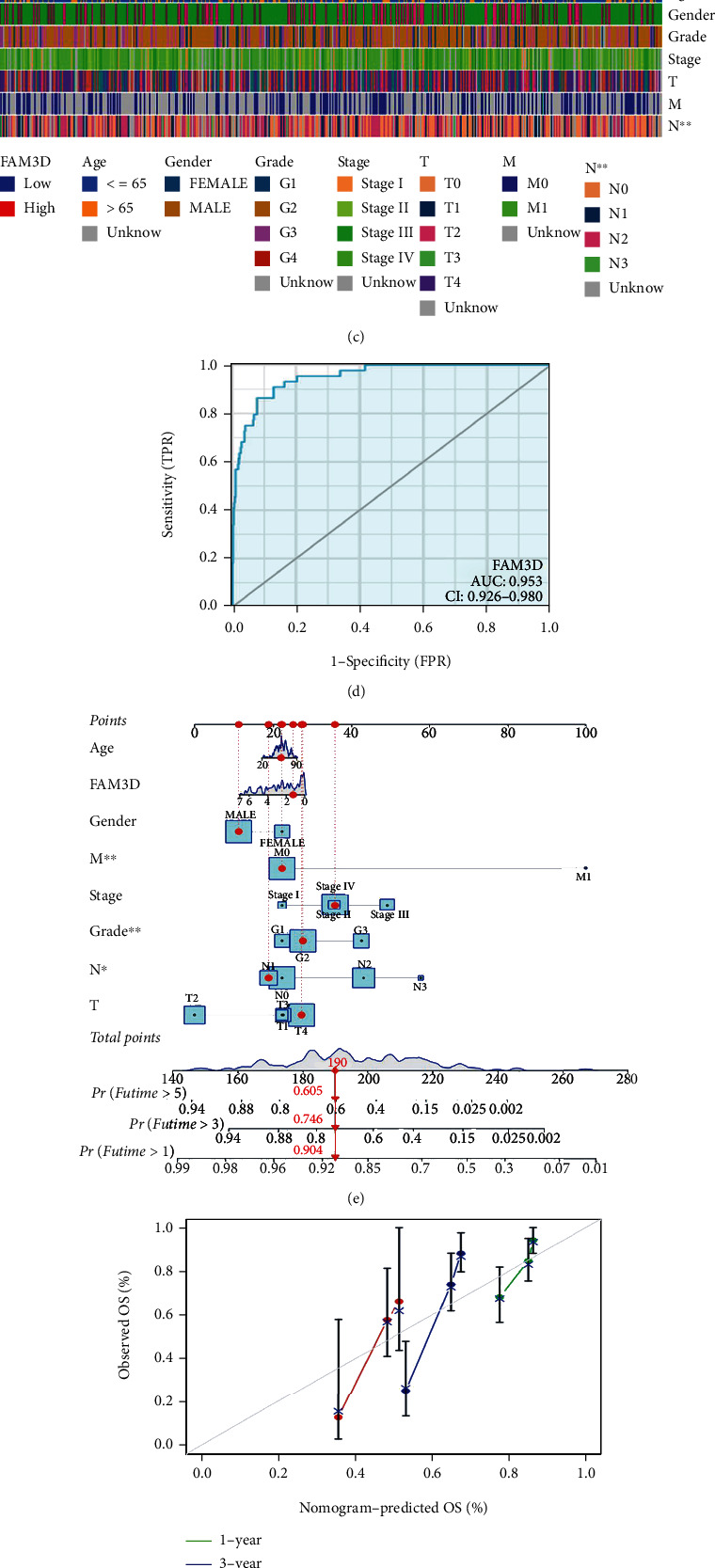
Association of *FAM3D* with clinical factors and prognosis in TCGA cohort. (a–c) *FAM3D* expression in HNSCC samples with advanced T and N stages. (d) Diagnostic values of *FAM3D* were shown in ROC curves. (e) Calibration plots validated the efficiency of nomograms for OS. (f) The nomogram was performed to establish *FAM3D* expression-based risk scoring models for 1-, 3-, and 5-year overall survival. (g, h) Univariate and multivariate regression analyses.

*FAM3D* expression in HNSCC samples with advanced T and N stages was observed to be at a lower level than those with early T and N stages (Figures 5(a) and 5(b)). *FAM3D* expressions and clinical factors were also displayed in the heat map (Figure 5(c)). The ROC curve was visualized to identify the clinical value of *FAM3D* evaluation. Since the AUC was 0.953 (Figure 5(d)), *FAM3D* was highly feasible in diagnosing HNSCC. Age, gender, grade, *FAM3D*, clinical stage, T stage, N stage, and M stage were then evaluated to develop a prognostic nomogram, and the calibration curve was protracted to test the efficiency. It had a C-index of 0.904, 0.746, and 0.605 for the 1-, 3-, and 5-year survival rates, respectively (Figure 5(e)). The calibration curves demonstrated advisable prediction of the three nomograms for the 1-, 3-, and 5-year clinical effectiveness values (Figure 5(f)). The univariate regression analysis indicated that *FAM3D* was a favorable prognostic factor, while the multivariate regression analysis indicated that *FAM3D* served as an independent prognostic factor (Figures 5(g) and 5(h)).

### 3.4. Correlation between the Expression Level of *FAM3D* and Clinicopathological Features in the GEO Cohort and the CPTAC Database Samples

**Figure 6:**
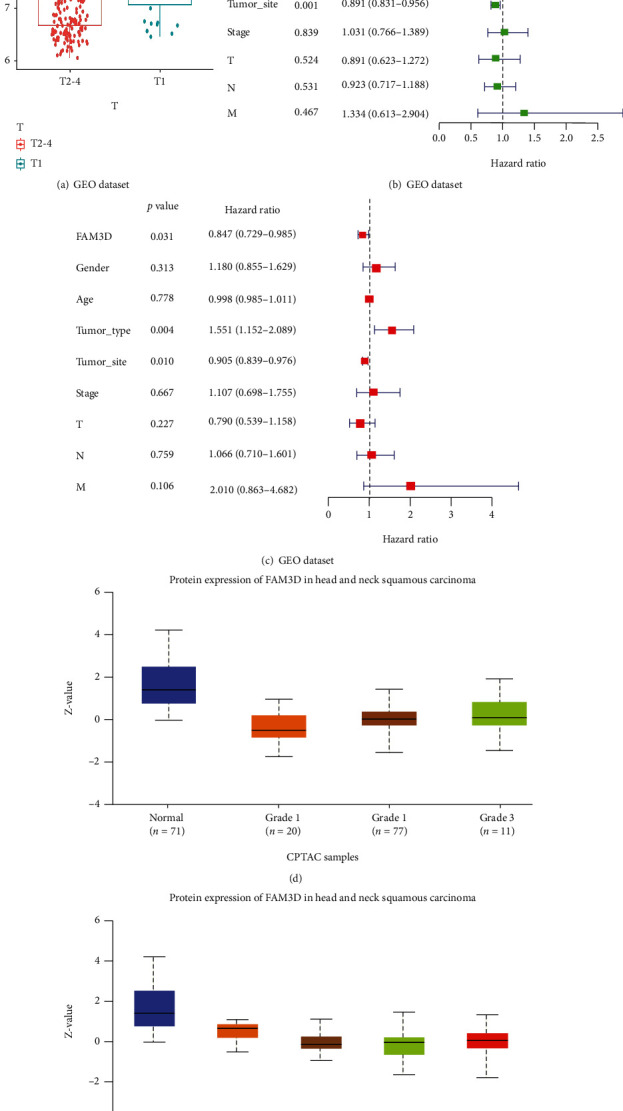
Correlation between the expression level of *FAM3D* and the clinicopathological features in the GEO and CPTAC datasets. (a) Correlation between *FAM3D* gene expression and T stage in the GEO cohort. (b, c) Univariate and multivariate regression of *FAM3D* in HNSCC in the GEO cohort. (d, e) Correlation between *FAM3D* protein expression and grade and stage in the CPTAC dataset.

Acts as an independent dataset for external validation, in the GEO cohort, *FAM3D* expression levels were inversely correlated with the T stage (Figure 6(a)). Univariate regression analysis and multivariate regression analysis indicated that *FAM3D* served as an independent favorable prognostic factor (Figures 6(b) and 6(c)). In the CPTAC samples, the expression of the *FAM3D* protein tends to descend with the increase of grade and stage (Figures 6(d) and 6(e)).

### 3.5. Coexpressed Genes of *FAM3D* in HNSCC Specimens in TCGA Cohort

**Figure 7:**
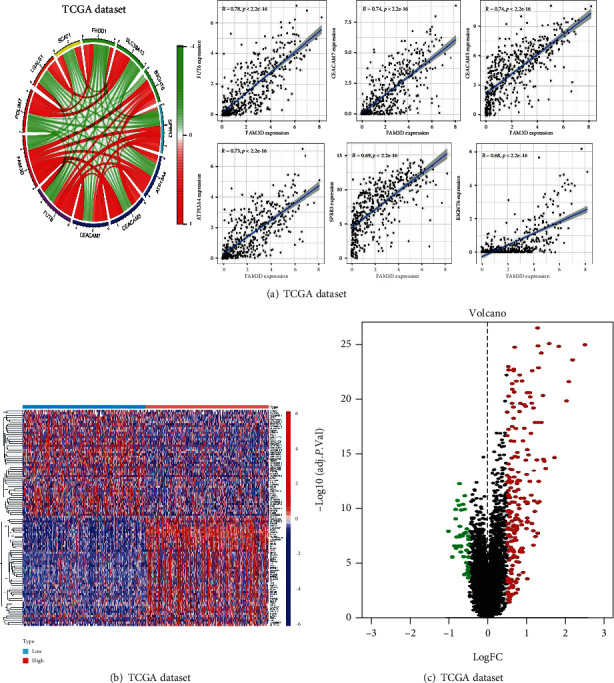
Coexpressed and DEGs of *FAM3D* in HNSCC specimens in TCGA cohort. (a) Coexpressed genes of *FAM3D*. (b, c) Heat map and volcano map showing DEGs in the *FAM3D* high- and low-expression groups.

In order to further study the mechanism of the effect of *FAM3D* on the prognosis of HNSCC, we explored the genes that interact with *FAM3D* in HNSCC. The circle graph showed that *FAM3D* expression was positively associated with *FUT6*, *CEACAM7*, *CEACAM5*, *ATP13A4*, *SPRR3*, and *B3GNT6* (Figure 7(a)). This study discovered 706 DEGs in the two subgroups (high- and low-*FAM3D*-expression). The heat map showed the top 50 downregulated or upregulated genes (Figure 7(b)). Red represented the upregulated genes, and green represented the downregulated genes in the volcano map (Figure 7(c)).

### 3.6. Functional and Pathway Enrichment Analysis

**Figure 8:**
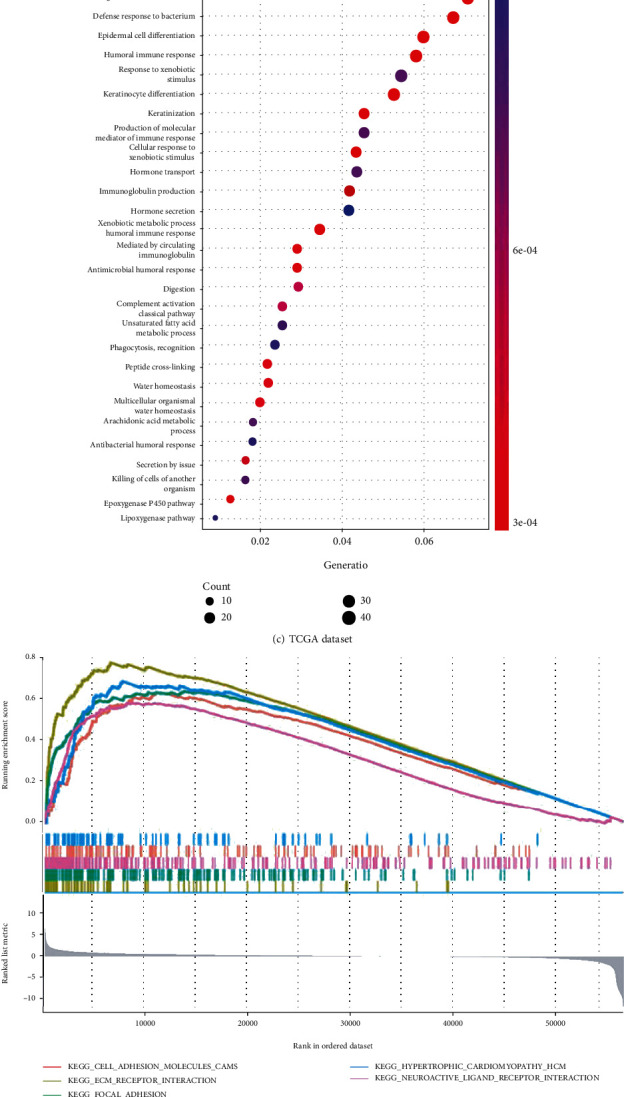
Functional and pathway enrichment analysis. (a–d) GO analysis, KEGG analysis, and GSEA in TCGA dataset. (e–i) Association between the expression of the *FAM3D* protein and the mTOR, MYC/MYCN, NRF2, HIPPO, and RTK pathways in the CPTAC dataset.

To further elucidate the biological impact of *FAM3D* on HNSCC, this study performed a GO analysis, a KEGG analysis, and GSEA. The GO analysis showed that there were more abundant immune-related molecular functions enrichment (Figures 8(a) and 8(b)). Similarly, the KEGG assays displayed that the DEGs were significantly enriched in the humoral immune response, in immunoglobulin production, and in the production of molecular mediators of the immune response (Figure 8(c)). The GSEA showed enrichment in pathways involving cell adhesion molecules (CAMs), receptor interaction, and focal adhesion (Figure 8(d)). In the CPTAC samples, the expression of *FAM3D* proteins in HNSCC tissues was statistically downregulated in the following pathways: mTOR, MYC/MYCN, NRF2, HIPPO, and RTK (Figures 8(e)–8(i)).

### 3.7. Immunological Properties of *FAM3D* in HNSCC Tissues in TIMER2 and TCGA

**Figure 9:**
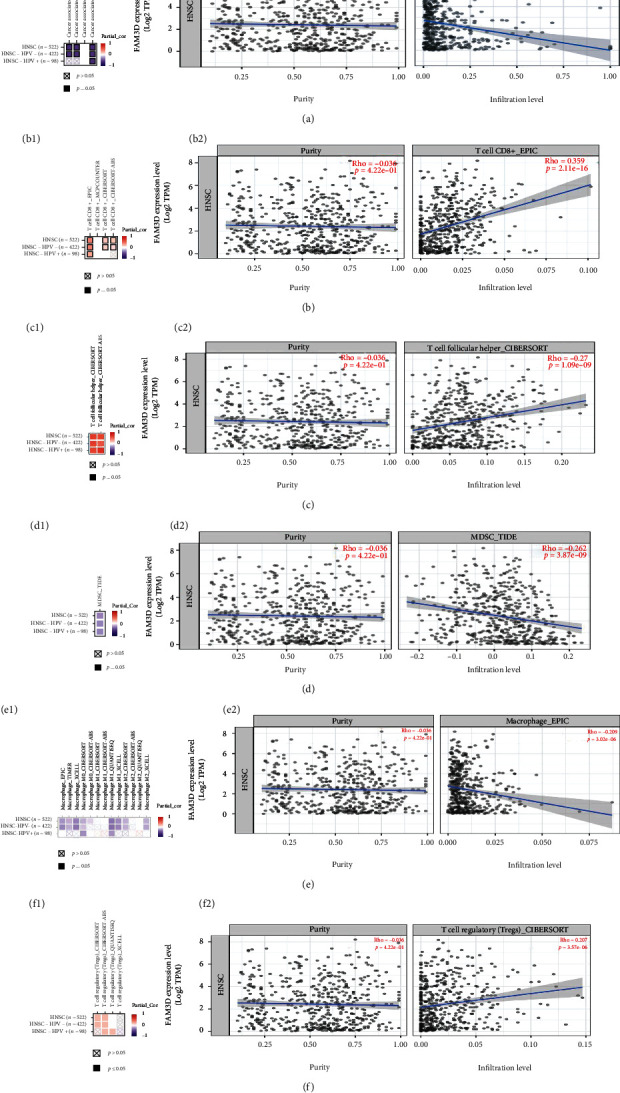
Immune infiltration analysis of *FAM3D* in HNSCC tissues. (a1, a2) Correlation between CAF immune infiltration and *FAM3D* expression in HNSCC. (b1, b2) Correlation between T cell CD8+ cells immune infiltration and *FAM3D* expression in HNSCC. (c1, c2) Correlation between Tfh cells immune infiltration and *FAM3D* expression in HNSCC. (d1, d2) Correlation between MDSC immune infiltration and *FAM3D* expression in HNSCC. (e1, e2) Correlation between macrophage cells, immune infiltration, and *FAM3D* expression in HNSCC. (f1, f2) Correlation between regulatory T cells, immune infiltration, and *FAM3D* expression in HNSCC.

We found that *FAM3D* was enriched in immune-related pathways in HNSCC, so next we studied the relationship between *FAM3D* and immune cell infiltration. In the TIMER2 model, the infiltration of CAFs, MDSCs, and macrophages in HNSCC tissues was inversely linked with *FAM3D* expression (Figures 9(a1) and (a2), 9(d1) and (d2), and 9(e1) and (e2)). Results showed that *FAM3D* expression level was positively correlated to T cell CD8+ cells, Tfh cell infiltration, and Treg cells and values (Figures 9(b1) and (b2), 9(c1) and (c2), and 9(f1) and (f2)).

**Figure 10:**
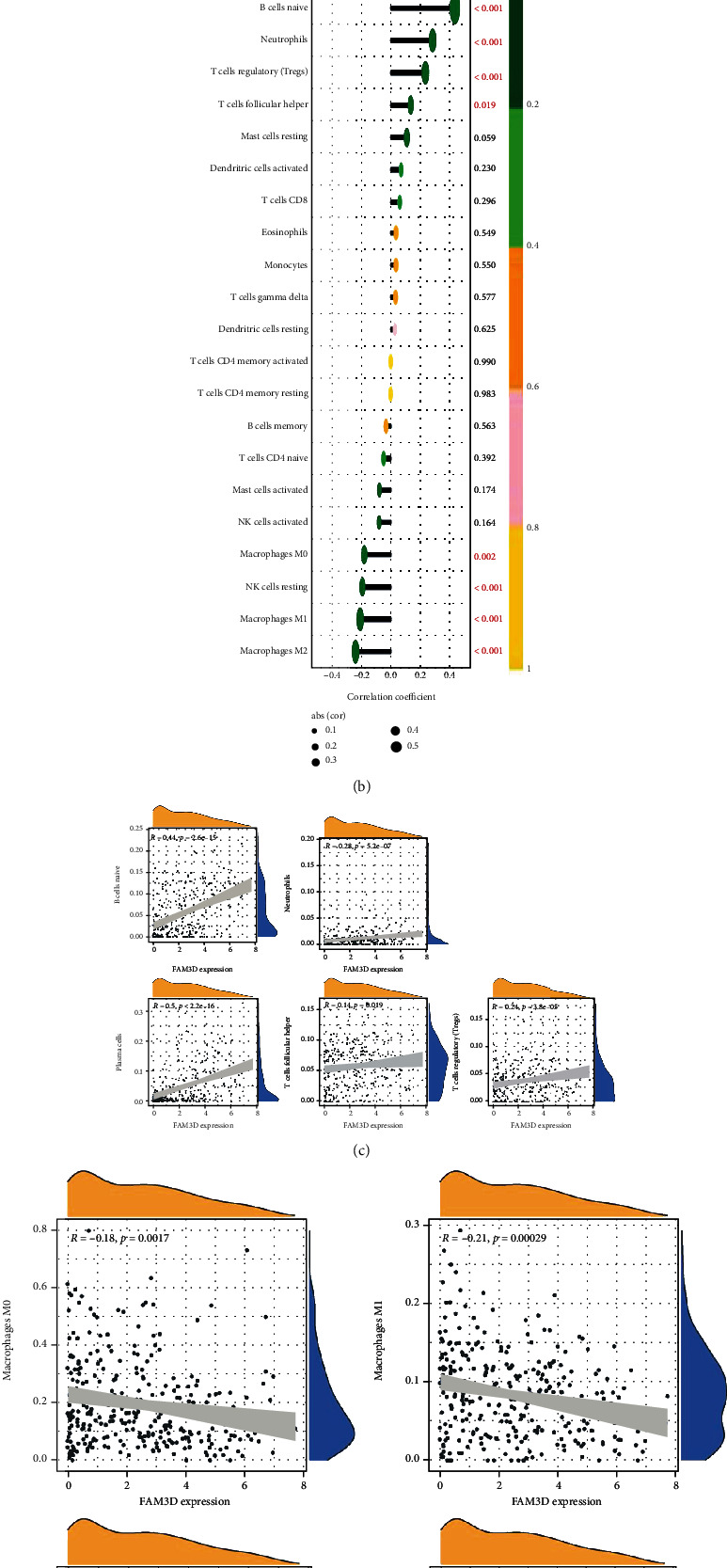
Correlations of *FAM3D* expressions with immune infiltration levels in the HNSCC tissues in TCGA cohort. (a) Determination of differential immune cells between the low- and high-*FAM3D*-expression groups. (b–d) Correlation between *FAM3D* and infiltrating immune cells in HNSCC tissues. ^∗^*p* < 0.05, ^∗∗^*p* < 0.01, and ^∗∗∗^*p* < 0.001.

In the TCGA cohort, the CIBERSORT algorithm was performed to study the 22 different types of immune cell profiles in the HNSCC samples. The differential infiltration of B naive cells, plasma cells, Tfhs, Treg cells, neutrophils, NK resting cells, macrophages (M0 cells), macrophages (M1 cells), and macrophages (M2 cells) in the two subgroups is presented in Figure 10(a). The results indicated that *FAM3D* was positively correlated with B naive cells, plasma cells, Tfhs, Treg cells, and neutrophils, while it was negatively correlated with NK resting cells, M0 cells, M1 cells, and M2 cells (Figures 10(b)–10(d)).

**Figure 11:**
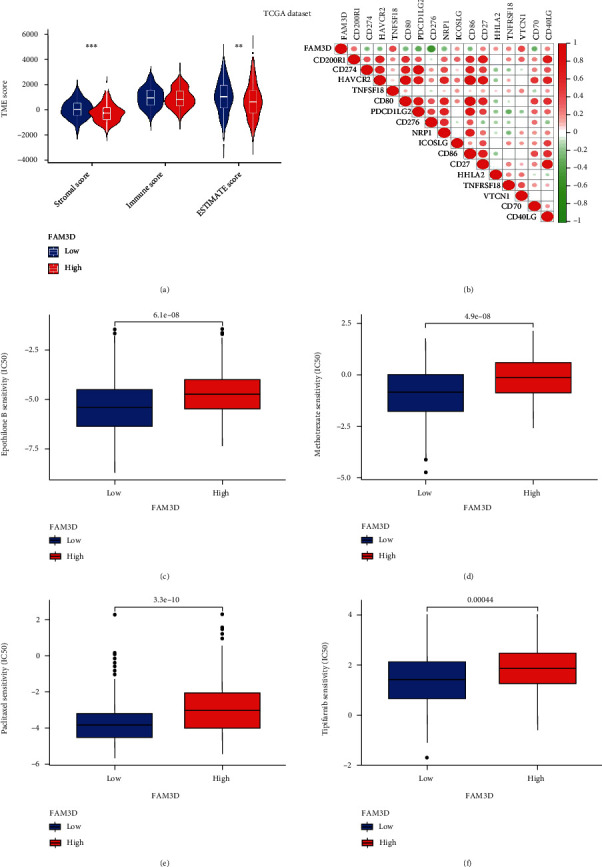
TME score, immune checkpoints, and drug sensitivity analysis of the *FAM3D* gene in HNSCC samples in TCGA cohort. (a) TME scores analysis in the high- and low-*FAM3D*-expression groups. (b) Correlations of *FAM3D* expressions with immune checkpoints. (c–f) Sensitivity of anticancer drugs in the high- and low-*FAM3D-*expression groups.

Then, we further investigated the immune microenvironment of HNSCC by ESTIMATE. High *FAM3D* level group showed higher stromal and ESTIMATE scores (*p* < 0.05), indicating that patients with a low level of *FAM3D* are more prone to an immune reaction (Figure 11(a)). Expressions of immune checkpoints and their ligands may be predictive biomarkers for immune checkpoint blockade therapy. The relationships between 16 immune checkpoint inhibitors and *FAM3D* (Figure 11(b)) were investigated; the results revealed that positive associations existed between the expression of *FAM3D* and the expressions of the following immune checkpoints: CD200R1, TNFRSF18, ICOSLG, CD27, HHLA2, TNFSF18, VTCN1, and CD40LG. In addition, the expression of *FAM3D* was negatively linked with CTLA4, HAVCR2, CD80, PDCDILG2, CD276, NRP1, CD86, and CD70.

### 3.8. Drug Sensitivity Analysis of the *FAM3D* Gene

To identify the efficacy of *FAM3D* as biomarkers for predicting the treatment effectiveness in patients with HNSCC, the IC50 values for common anticancer drugs were explored with the pRRophetic algorithm in the *FAM3D* high- and low-expression groups. The drugs included medications approved by the United States FDA and clinically validated substances that have been used to treat patients with HNSCC. Research indicated that people with high-*FAM3D*-expression were more sensitive to epothilone B, methotrexate, paclitaxel, and tipifarnib (Figures 11(c)–11(f)). The observation hinted that *FAM3D* may exert action in HNSCC's sensitivity to antitumor medications.

## 4. Discussion

The substantial morbidity and mortality rates associated with HNSCC have sparked interest in developing biomarkers for the early detection and accurate prognosis prediction of the disease. However, no commonly recognized biomarkers for HNSCC have been identified. As a result, it is critical to investigate novel useful biomarkers for HNSCC.

In our study, lower *FAM3D* mRNA and protein expression were detected in HNHCC and were proved to be an adverse prognostic factor of HNHCC, which was externally verified by different databases. The results were also consistent with previous studies. *FAM3D* deficiency can promote the incidence and development of colon cancer [27]. Zhou et al. identified downregulation of the *FAM3D* gene in nasopharyngeal cancer tissue specimens [28]. It was discovered that the level of *FAM3D* expression in HNSCC tissues was highly linked with clinicopathological characteristics such as T grade, stage, and lymph node metastasis. We found that *FAM3D* acted as an independent favorable prognostic factor in HNSCC. Furthermore, the ability to utilize *FAM3D* in predicting HNSCC prognosis was validated internally in the TCGA cohort. When integrating *FAM3D* expression, age, and stage, the stability and reliability of the nomogram in predicting the 1-, 3-, and 5-year OS in patients with HNSCC were confirmed. The predictive value of such a prognostic model is also reflected in other studies [29]. These findings imply that *FAM3D* may provide a novel target for the treatment and prognosis prediction of patients with HNSCC.

Our research suggested that *FAM3D* expression was positively correlated with these genes: *FUT6*, *CEACAM5*, and *SPRR3*. *FUT6* was significantly downregulated in malignant tissues in comparison with their adjacent normal tissues [30]. Overexpression of *FUT6* inhibits the malignant activity of tumor cells by inhibiting the dimerization and phosphorylation of the epidermal growth factor receptor [31]. CEACAM5 is a cell surface glycoprotein that is usually expressed on the epithelial cells in many organ systems [32]. CEACAM5 was downregulated in most HNSCC tissues compared with the adjacent normal tissues [33, 34]. This finding indicates that for CEACAM5, overexpression may inhibit the occurrence and development of HNSCC. *SPRR3* was identified as a novel diagnostic/prognostic biomarker for oral squamous cell carcinoma (OSCC), and its low expression predicted an unfavorable prognosis in patients with OSCC [35]. *FAM3D* was coexpressed with these genes. But, as a favorable gene, the protective mechanism of *FADM3D* in HNSCC requires further investigation.

To research the potential function of *FAM3D* in the development of HNCC, a GO analysis, a KEGG assay, and GSEA were performed using genes dysregulated between the HNSCC samples with both high- and low-*FAM3D*-expression. The activity of many immune-related molecules was significantly enhanced in the GO analysis. The KEGG analysis still found a significant enrichment in immunoactive pathways. This study also explored the relationship between FAM3D protein expression and pathways such as mTOR, MYC/MYCN, and NRF2 in HNSCC tissues. The group that altered these pathways had downregulated FAM3D protein expression compared with the normal group. mTOR can regulate the function of dendritic cells and plays an important role in the activation of T cells and the function of Treg cells. Immune cells were found to modulate host responses by using mTOR receptors that signal either directly or indirectly [36]. Studies have shown that the small-molecule MYC inhibits tumor growth and enhances immunotherapy. MYC inhibitors induce immunogenic cell death in tumor cells and cause increased T cell infiltration and subsequently upregulate PD-L1 in the tumor microenvironment [37]. NRF2 expression in the host has been reported to limit tumor growth by maintaining a functional immune system, while NRF2 in cancer cells promotes tumor growth [38]. Therefore, this study's findings indicated that *FAM3D* takes part in the altered immune status of HNSCC.

By GESA of *FAM3D* in HNSCC, we found that there was a close link between *FAM3D* expression and immune status. Then, we searched its functions in TME. TME was composed of tumor cells, various immune infiltrating cells, stromal cells, and cytokines [39]. Tumor-infiltrating immune cells, as a critical component of the tumor microenvironment, are intimately linked to the incidence, progression, and metastasis of tumors [40, 41]; additionally, CAFs have been found in the stroma of the TME and likely control the function of a variety of tumor-infiltrating immune cells [42]. The activation of cMet by CAFs via paracrine hepatocyte growth factor has shown to be a contributory event in HNSCC progression [43]. There was a statistically significant negative connection between the immune infiltration of CAFs and *FAM3D* expression levels in HNSCC tissues in this study. Additionally, increased macrophages are associated with a poor prognosis because they play a key role in innate immunity [44, 45]. Gao et al. discovered that a positive feedback loop between cancer cells and macrophages can increase HNSCC cell migration and invasion [46]. A systematic review and meta-analysis confirmed the prognostic benefit of CD8+ T cell infiltration in HNSCC patients and found that FoxP3+ TIL contributed to improve OS [47]. Previous studies have also shown that the Treg/CD8+ T cell ratio is associated with a higher survival rate for HNSCC patients [48] and that the increase of CD8+ TILs is a good independent predictor [49]. Study showed that a high Treg count was associated with a better prognosis for patients with HNSCC [50]. According to this study's findings, increased expression of *FAM3D* may limit macrophage and MDSC infiltration into the tumor's immune environment while promoting the infiltration of T cell CD8+ cells, Treg cells, and Tfh cells. This study provided more evidence that *FAM3D* is a good prognostic factor in HNSCC. We found the stroma score; the estimated score was significantly higher in the low-*FAM3D*-expression group when compared with the high-*FAM3D*-expression group. This indicated that *FAM3D* may participate in the tumor genesis and progression by affecting the TME. The prognostic features of *FAM3D* were significantly associated with immune cell infiltration, which may play a role in both immunotherapy and targeted therapy in patients with HNSCC. However, this needs confirmation in future studies.

Immunotherapy has revolutionized the pattern of cancer treatment over the past decade by strengthening patients' own immune systems to remove cancer cells. The immune checkpoints of tumor cells are usually so activated that the antigen cannot be presented to T cells, and the normal immune function of T cells is suppressed. With the development of drugs that target tumor immune checkpoints, immunoblocking therapies have become a new anticancer weapon. Nivolumab and pembrolizumab have been approved for the treatment of advanced HNSCC [51, 52]. However, immunotherapy has a substantial benefit for only a small subset of cancer patients. The clinical response rate of nivolumab and pembrolizumab monotherapy in Asian patients was only one in five according to the available data [53]. In this study, the different associations that existed between the expression of *FAM3D* and the immune checkpoints were detected, which may provide clues for the targeted immunotherapy of *FAM3D* in HNSCC.

Since chemotherapy remains a key treatment strategy for HNSCC, we developed a chemotherapeutic drug sensitivity analysis of patients in the high-*FAM3D*-expression group and the low-*FAM3D*-expression group in view of the GDSC database. Results showed that high-*FAM3D*-expression was accompanied by the hyperreactivity of these approved drugs, such as epothilone B, methotrexate, paclitaxel, and tipifarnib. However, chemotherapy has limited efficiency and effective duration. The combination of chemotherapy and immunotherapy provides a new treatment model for patients with HNSCC [54]. Therefore, it is important to further explore the treatment modalities and mechanisms for targeting *FAM3D* in HNSCC.

This study did have some limitations. First, this study was retrospective. Therefore, future prospective studies with larger sample sizes are needed to confirm these findings. Second, we confirmed the expression of *FAM3D* in HNSCC experimentally, but the underlying biological mechanisms require further investigation.

## 5. Conclusion

In this study, the *FAM3D* was a valuable prognostic biomarker for HNSCC. And its association with immune infiltrate levels could help to further stratify HNSCC patients who will benefit more from immunotherapy.

## Data Availability

All data generated or analyzed during this study are included in this published article.

## Supplementary Materials

Supplementary Materials We thank Medjaden Inc. for the scientific editing of this manuscript.
