# Supplementary material for: FAM3D as a Prognostic Indicator of Head and Neck Squamous Cell Carcinoma Is Associated with Immune Infiltration
Source: Comput Math Methods Med. 2022 Dec 3;2022:5851755. doi: 10.1155/2022/5851755 (PMC9741545; doi:10.1155/2022/5851755)
Supplement: Supplementary Materials — We thank Medjaden Inc. for the scientific editing of this manuscript. [file 5851755.f1.pdf]

Sep15th., 2022

Language Certificate

Dear Sir or Madam:

This document certifies that the manuscript **MJD2209044**:

FAM3D, as a prognostic indicator of head and neck squamous carcinoma cells, is associated with immune infiltration(prepared by the authors: Lizhu Chen, et al.)

was edited for proper English language, grammar, punctuation, spelling, and overall style by a highly qualified, native English-speaking professional scientific editor at Medjaden Inc. on Sep15th., 2022. Neither the research content nor the authors' intentions were altered in any way during the editing process. The final revised version can be found at http://pan.medjaden.com:2000/index.php?mod=shares&sid=ZWFtREVQYlZ2YXhxREtlSGxTWWluOFkwc3lTQXk1LVZTTEI2am1z. (verification code: **WSJCLZ9044**)

Yours sincerely,

(Ms.) Hua (Selin) He for Medjaden Inc.

Information about Medjaden Inc.
Medjaden Inc. is an emerging editing company that provides professional services for laboratory researchers, postgraduates, and clinical doctors. Our mission is to help our clients initiate, maximize and accelerate the production of articles from their research work. The company employs experienced medical statisticians and editors drawn from English backgrounds worldwide, who strive to ensure that every edited article is published in an English journal, preferably one that is included in the Web of Science (WOS), the Science Citation Index (SCI) Expanded, Medline, PubMed, EMBASE, and other scientific resources.
Email: medjaden@gmail.com; Website: www.medjaden.com
